# Beyond 72 Hours: A Retrospective Evaluation of Factors Associated With Delayed Surgical Stabilization of Rib Fractures

**DOI:** 10.7759/cureus.112195

**Published:** 2026-07-07

**Authors:** Alexis A Schweibinz, Clarisse Solis, Anna Michaud, John Dillis, Katherine A Graham, William B DeVoe

**Affiliations:** 1 Department of Surgery, Riverside Methodist Hospital (OhioHealth), Columbus, USA; 2 Department of Trauma, Riverside Methodist Hospital (OhioHealth), Columbus, USA; 3 Department of Surgery, OhioHealth Research Institute, Columbus, USA

**Keywords:** chest wall injury, flail chest, rib fixation, rib fractures, surgical stabilization of rib fractures(ssrf)

## Abstract

Background: Surgical stabilization of rib fractures (SSRF) is recognized as a key intervention in reducing morbidity among patients with multiple displaced rib fractures or flail chest. Early SSRF, typically within 72 hours of admission, has been associated with improved outcomes. The factors contributing to delayed stabilization, defined as 72 hours or greater following admission, remain unclear.

Methods: A retrospective cohort study was performed, evaluating 492 trauma patients who underwent SSRF at a level II trauma center. Patient demographics, injury characteristics, and associated injuries were analyzed to identify factors associated with delayed SSRF. Groups were defined as early (≤72 hours) and delayed (>72 hours). Regression analysis was used to identify independent predictors of delay. Correlations between time to operation and hospital outcomes were evaluated. A p-value of <0.05 was considered significant.

Results: Of 492 patients, the median age was 66 years, and 301 (61%) were male; 122 (24.8%) patients underwent SSRF beyond 72 hours from admission. On univariate analysis, there was no difference in age, number of rib fractures, presence of flail chest, or anticoagulation status between groups. Delayed intervention was associated with higher ISS (19 vs 15, p<0.001), lower admission GCS (p<0.001), and need for craniotomy (p=0.002). On multivariate analysis, only injury severity score (ISS) independently predicted surgical delay (p=0.002, CI 1.01-1.07). Delayed SSRF correlated with longer hospital stay (p<0.001) but not ICU stay (p=0.876). Inpatient mortality did not differ significantly between early and delayed groups (1.6% vs. 4.1%, p=0.110).

Conclusions: Following a retrospective review of nearly 500 patients undergoing SSRF for severe flail and non-flail chest wall injury at a single institution, the ISS was found to be associated with delayed intervention beyond 72 hours of admission. Despite the known potential for increased risk of delayed chest wall stabilization, ICU stay, and inpatient mortality were found to be similar between early and delayed SSRF.

## Introduction

Rib fractures are a frequent consequence of blunt thoracic trauma and are associated with significant morbidity, particularly in patients with multiple displaced rib fractures or flail chest [1]. Effective management requires a multifaceted approach focused on pain control, pulmonary optimization, and, in select cases, surgical stabilization. Over the past two decades, there has been increasing evidence to support surgical stabilization of rib fractures (SSRF) in appropriately selected patients for both flail and severe non-flail chest wall injuries [2-4]. The SSRF has been shown to reduce ventilator days, hospital length of stay, and mortality [5-12]. The morbidity of severe chest wall injury is particularly detrimental in the frail and elderly, with this at-risk patient population also benefiting from chest wall stabilization [13-15].

While early SSRF, typically within 72 hours of admission, has been associated with improved outcomes and reduced complications, the factors influencing the timing of surgery remain poorly understood [16-18]. In clinical practice, delayed SSRF is often a reflection of physiologic instability, concomitant injuries requiring prioritization, or logistical constraints. The factors that contribute to delayed stabilization remain incompletely defined [19].

A better understanding of elements that contribute to delayed SSRF is central to optimizing surgical decision-making and ensuring that appropriate patients receive timely stabilization when feasible. In this study, we sought to better define the clinical and injury-related factors associated with delayed operative fixation of rib fractures. Additionally, we aimed to evaluate the relationship between delayed stabilization and key clinical outcomes. The investigation aimed to improve understanding of factors influencing surgical timing and identify potential areas for process improvement within our institution.

## Materials and methods

Study design

A retrospective review was performed of adult trauma patients who underwent SSRF at a level II trauma center. Approval was obtained from the OhioHealth Office of Human Subjects Protection and Institutional Review Board with a waiver of informed consent and Health Insurance Portability and Accountability Act of 1996 (HIPAA) authorization requirements, given the retrospective nature of the study posing minimal risk to the subjects. Patients who underwent SSRF between January 1, 2016, and October 31, 2024, were identified through the electronic medical record and institutional trauma registry. Imaging (plain radiographs, CT imaging), operative notes, clinical documentation, laboratory results, and discharge summaries were reviewed to confirm SSRF.

Data collection

Patient demographic and clinical information, including age, injury severity score (ISS), chest abbreviated injury scale (AIS), Glasgow Coma Scale (GCS), number of rib fractures, presence of flail chest, bilateral chest wall injury, and anticoagulation/antiplatelet status, was collected [20]. In addition, in-hospital outcomes such as time to SSRF, need for major operations (spine, brain, femur, and pelvis), ICU length of stay, hospital length of stay, and inpatient mortality were noted.

Inclusion and exclusion criteria

All patients aged 18 years or older undergoing SSRF between January 1, 2016, and October 31, 2024, were included. Patients under the age of 18 were excluded. Patients were also excluded when the time to operating room data could not be obtained. Patients requiring SSRF for injuries related to cardiopulmonary resuscitation (non-traumatic) were also excluded.

Outcomes

The primary aim was to identify demographic and clinical factors that place patients at risk for delayed SSRF. This measure was examined using clinically relevant thresholds of 72 hours or more, based on prior evidence suggesting that prolonged time to operation may negatively influence clinical outcomes. Secondary outcomes were selected to assess the association between time to OR, ICU length of stay, hospital length of stay, and inpatient mortality. Both ICU and hospital length of stay were measured in days. Inpatient mortality was defined as any death occurring during the patient’s hospitalization. 

Statistical analysis

Statistical analysis was performed using Jamovi version 2.3.26.0 (Jamovi Project, Sydney, AUS). Continuous variables were assessed for normality and summarized with appropriate descriptive statistics, while categorical variables were reported as counts and percentages. Univariate analyses using Mann-Whitney U (continuous variables) and chi-squared (categorical variables) tests were conducted as appropriate to examine associations between time to SSRF and demographic, clinical, and surgical variables, as well as relationships with ICU length of stay, hospital length of stay, and inpatient mortality. A regression analysis was performed following the univariate analysis to identify discrete factors contributing to delayed fixation. Statistical significance was defined as p < 0.05.

## Results

A total of 492 patients underwent SSRF during the study period. The median age was 66 years (range 19-100), and 301 (61%) patients were male (Table 1). Around 98% of patients received SSRF within seven days (n=484). Table 2 demonstrates the comparison between early and delayed fixation cohorts; 370 (75.2%) patients underwent early fixation, with 122 (24.8%) patients undergoing delayed fixation during the study period. The median number of rib fractures was similar between early (six fractures, range one to 22) and delayed (six fractures, range two to 15) groups (p=0.126). Presence of flail chest anatomy was similar between early and delayed fixation groups (161 (43.6%) early vs. 51 (42.5%) late, p=0.828). Bilateral chest wall injury also was comparable between the cohorts (46 (12.5%) early vs. 19 (15.6%) delayed, p=0.380). Similar proportions of patients on anticoagulation (41 (11.1%) early vs. 17 (13.9%) delayed, p=0.397) and antiplatelet therapy (114 (30.8%) early vs. 37 (30.3%) delayed, p=0.920) were also found between early and delayed fixation. On univariate analysis, delayed SSRF was associated with higher ISS (median 19 vs. 15, p<0.001), higher chest AIS (p=.003), and lower GCS on arrival (p=0.001). Only 2.5% of patients received a major brain operation and were also more likely to experience delay (p=0.002).

**Table 1:** Patient demographics (total n =492)

Characteristics		Value
Age, median (range)		66 (19-100)
Gender, n (%)	Female	191 (38.8)
	Male	301 (61.2)

**Table 2:** Factors associated with delayed SSRF Mann-Whitney U tests were used for age, ISS, chest AIS, GCS on arrival, and number of rib fractures. Chi-squared tests were used for the remaining variables. SSRF: Surgical stabilization of rib fractures, ISS: Injury Severity Score, AIS: Abbreviated Injury Scale, GCS: Glasgow Coma Scale

Variable	Early SSRF (n=370)	Delayed SSRF (n=122)	p-value
Age, median (range)	66 (22-100)	63.50 (19-94)	0.627
ISS, median (range)	15 (2-57)	19 (4-50)	<0.001
Chest AIS, median (range)	3 (1-5)	3 (1-5)	0.003
GCS on arrival, median (range)	15 (3-15)	15 (3-15)	0.001
Number of rib fractures, median (range)	6 (1-22)	6 (2-15)	0.126
Presence of flail chest, n(%)	161 (43.63)	51 (42.50)	0.828
Bilateral chest wall injury, n(%)	46 (12.47)	19 (15.57)	0.380
Need for major spine operation, n(%)	13 (3.51)	8 (6.56)	0.149
Need for major brain operation, n(%)	0 (0.00)	3 (2.46)	0.002
Need for major femur operation, n(%)	10 (2.70)	5 (4.10)	0.437
Need for Major Pelvis Operation, n(%)	9 (2.43)	5 (4.10)	0.337
Anticoagulation, n(%)	41 (11.08)	17 (13.93)	0.397
Antiplatelet, n(%)	114 (30.81)	37 (30.33)	0.920

On multivariate analysis, ISS was the only independent predictor of surgical delay (OR 1.04, 95% CI 1.02-1.07, p=.001). Chest AIS was excluded from the regression analysis, as the chest wall injury burden based on the number of rib fractures and the presence of flail chest were similar between the groups. Both a logistic regression analysis using binary groups categorized by the set time threshold of 72 hours (i.e., 'early' and 'delayed' groups) and a linear regression analysis using time to OR as a continuous variable were performed (Tables 3-4). The need for major brain, spine, pelvis, or femur operations did not independently predict delay. The need for a major brain operation was excluded from the linear regression analysis given that only three patients underwent craniotomy and SSRF. Not unexpectedly, of the three patients undergoing both craniotomy and chest wall stabilization, the average time to SSRF was 208 hours. Time to operation correlated with hospital length of stay (ρ=0.40, p<0.001) but not the ICU stay (p=0.868) (Table 5). Inpatient mortality was not significantly different between early and delayed groups (1.6% vs. 4.1%, p=0.110).

**Table 3:** Multivariable logistic regression of factors associated with delayed SSRF *Only three patients in the data set required a brain operation. SSRF: Surgical stabilization of rib fractures, ISS: Injury Severity Score, GCS: Glasgow Coma Scale

Factors	OR (95% CI)	p-value
ISS	1.04 (1.02-1.07)	0.001
GCS on arrival	0.94 (0.85-1.04)	0.232
Need for major brain operation	0.00 (0.00-Inf)*	0.978

**Table 4:** Multivariable linear regression of factors associated with delayed SSRF Patients requiring a major brain operation were excluded from continuous linear regression (n=3). SSRF: Surgical stabilization of rib fractures, ISS: Injury Severity Score, GCS: Glasgow Coma Scale

Factors	Standard estimate (95% CI)	p-value
ISS	0.16 (0.07-0.25)	<0.001
GCS on arrival	-0.09 (-0.18-0.01)	0.072
Need for major spine operation	-0.37 (-0.78-0.05)	0.081
Need for major femur operation	-0.15 (-0.65-0.34)	0.543
Need for major pelvic operation	-0.31 (-0.82-0.21)	0.242

**Table 5:** Time to SSRF vs. ICU and hospital length of stay Spearman's rho measures the strength and direction of two variables from –1 to +1. A value near +1 indicates a strong positive relationship (as one increases, the other tends to increase), while a value near –1 shows a strong negative relationship (as one increases, the other tends to decrease). Values close to 0 indicate weak or no association. SSRF: Surgical stabilization of rib fractures

Outcome	Time to SSRF (Spearman’s rho)	p-value
ICU length of stay	-0.04	0.868
Hospital length of stay	0.40	<0.001

## Discussion

Rib fractures are often associated with substantial morbidity, particularly in patients with multiple displaced fractures or flail chest [1]. Surgical stabilization of rib fractures has become an accepted component of the management of severe chest wall injury, with growing evidence supporting improved outcomes and reduced pulmonary complications [2-12]. In addition to identifying appropriate candidates for repair, increasing attention has been directed toward the timing of SSRF, as early intervention has been associated with improved outcomes [16-18]. Despite these findings, the factors that influence the timing of operative fixation in clinical practice remain poorly understood [19].

In this retrospective cohort study of 492 trauma patients undergoing SSRF, the clinical factors associated with delayed operative stabilization were evaluated to determine the effects of this delay on patient outcomes. Nearly 25% of patients during the study period underwent SSRF beyond 72 hours after admission. Patients who experienced delay were more severely injured, as reflected by higher ISS and lower admission GCS scores, suggesting a greater overall injury burden. However, on multivariate analysis, ISS was the only independent predictor of delayed surgery. This was consistent both on logistic regression analysis comparing groups based on the 72-hour time threshold and on linear regression analysis using time to OR as a continuous variable. Despite this elevated injury burden, ICU length of stay and mortality did not differ significantly between early and delayed groups.

Much of the existing literature on SSRF timing has focused on comparing outcomes between early and delayed operative fixation, with many studies demonstrating a benefit when stabilization is performed within the first 72-82 hours after injury, as described by Pierraci et al. and Kwon et al. [16,18]. Consensus statements and guidelines from the World Society of Emergency Surgery (WSES), Chest Wall Injury Society (CWIS), and American Association for the Surgery of Trauma (AAST) recommend early stabilization of severe chest wall injuries, ideally within 48-72 hours of presentation [21-22]. The WSES-CWIS position paper and WSES-AAST thoracic trauma guidelines do comment on evaluating patients on a case-by-case basis for SSRF when other competing injuries or hemodynamic instability rightfully take priority in the initial days following presentation, while also potentially contributing to delayed chest wall stabilization. 

Prins et al. highlight that identification of discrete factors that may influence time to surgery for severe chest wall injury is limited at the current time in the literature [19]. Presumably, delays to surgery may represent modifiable system factors, patient characteristics, injury patterns, or appropriate prioritization of competing injuries in patients with multisystem trauma. The findings herein suggest that delayed SSRF is largely driven by overall injury severity rather than the presence of any single associated injury. Although patients with delayed stabilization were more likely to have lower admission GCS and require a major intracranial operation, these factors were not independently associated with delay after controlling for overall injury severity. The number of patients requiring craniotomy and SSRF was low, limiting the ability to draw definitive conclusions regarding the influence of a major intracranial procedure on the timing of chest wall stabilization. The total number of rib fractures, presence of flail chest anatomy, or bilateral chest wall injury appeared to directly influence time to fixation. Use of anticoagulation or antiplatelet medication also did not appear to impact time to surgery. Interestingly, the need for other major associated operations (spine, femur, and pelvis) was not an independent predictor of delay. This is likely attributable to multidisciplinary collaboration amongst surgical specialties, operating room access, and attempts to combine procedures when feasible.

Notably, delayed SSRF was not associated with increased inpatient mortality despite an elevated ISS as compared to the early fixation cohort. While early fixation has been associated with improved pulmonary and intraoperative outcomes in prior studies, our results suggest that delayed stabilization does not necessarily confer increased mortality when operative timing is guided by overall clinical stability. These findings support the safety of delayed fixation in appropriately selected trauma patients and emphasize the importance of individualized clinical decision-making when determining the optimal timing of SSRF. Delayed SSRF was associated with longer hospital length of stay but not increased ICU length of stay. Overall hospital LOS as an outcome relating to SSRF is limited by associated injuries and the often lengthy disposition process, which frequently occurs in trauma care. This difference further reflects the greater overall injury severity among patients undergoing delayed stabilization. As expected, patients with higher ISS frequently require prolonged hospitalization due to the cumulative impact of multisystem trauma and the need for extended recovery and rehabilitation.

Several limitations of this study should be acknowledged. First, this is a retrospective analysis from a single institution, which may limit generalizability to other trauma centers with different patient populations or institutional workflows. Second, despite the use of multivariable regression, residual confounding due to differences in baseline characteristics, such as injury severity score, cannot be entirely ruled out. In addition, other potential contributors to surgical delay, such as operating room availability, scheduling constraints, and institutional resource limitations, were not captured in the dataset. As previously discussed, the number of patients requiring craniotomy and chest wall stabilization was low, limiting the ability to draw definitive conclusions regarding the influence of a major intracranial procedure on the timing of SSRF. Finally, the use of a 72-hour threshold to define delayed SSRF reflects commonly used definitions in the literature but may not represent a strict physiologic boundary for optimal intervention.

## Conclusions

Patients undergoing delayed surgical stabilization of rib fractures demonstrated greater overall injury severity as reflected by higher ISS. Despite a higher injury burden and longer time to chest wall stabilization, ICU length of stay and inpatient mortality were similar between early and delayed groups. Although early SSRF is optimal, the timing of surgical intervention should be individualized to account for competing injuries and overall clinical stability. The findings herein support an individualized approach to chest wall stabilization in which delayed fixation reflects thoughtful coordination of care rather than a failure to achieve timely treatment. Delayed chest wall stabilization, when guided by multidisciplinary assessment and physiologic readiness, is a safe and appropriate strategy in complex, multiply injured trauma patients.
